# Oral manifestations, dental management, and a rare homozygous mutation of the *PRDM12* gene in a boy with hereditary sensory and autonomic neuropathy type VIII: a case report and review of the literature

**DOI:** 10.1186/s13256-017-1387-z

**Published:** 2017-08-15

**Authors:** Karim Elhennawy, Seif Reda, Christian Finke, Luitgard Graul-Neumann, Paul-Georg Jost-Brinkmann, Theodosia Bartzela

**Affiliations:** 1 Center for Dental and Craniofacial Sciences, Department of Orthodontics, Dentofacial Orthopedics and Pedodontics, Charité – Universitätsmedizin Berlin, Aßmannshauser Str. 4-6, 14197 Berlin, Germany; 2 Ambulantes Gesundheitszentrum, Campus Virchow Clinic, Charité – Universitätsmedizin Berlin, Berlin, Germany; 3 Charité Campus Virchow, Department of Human Genetics, Charité – Universitätsmedizin Berlin, Augustenburger Platz 1, 13353 Berlin, Germany

**Keywords:** Case report, Dental, *PRDM12* gene, Hereditary sensory and autonomic neuropathy, HSAN-VIII, Oral manifestations

## Abstract

**Background:**

Hereditary sensory and autonomic neuropathy type VIII is a rare autosomal recessive inherited disorder. Chen *et al*. recently identified the causative gene and characterized biallelic mutations in the PR domain-containing protein 12 gene, which plays a role in the development of pain-sensing nerve cells. Our patient’s family was included in Chen and colleagues’ study. We performed a literature review of the PubMed library (January 1985 to December 2016) on hereditary sensory and autonomic neuropathy type I to VIII genetic disorders and their orofacial manifestations. This case report is the first to describe the oral manifestations, and their treatment, of the recently discovered hereditary sensory and autonomic neuropathy type VIII in the medical and dental literature.

**Case presentation:**

We report on the oral manifestations and dental management of an 8-month-old white boy with hereditary sensory and autonomic neuropathy-VIII over a period of 16 years. Our patient was homozygous for a mutation of PR domain-containing protein 12 gene and was characterized by insensitivity to pain and thermal stimuli, self-mutilation behavior, reduced sweat and tear production, absence of corneal reflexes, and multiple skin and bone infections. Oral manifestations included premature loss of teeth, associated with dental traumata and self-mutilation, severe soft tissue injuries, dental caries and submucosal abscesses, hypomineralization of primary teeth, and mandibular osteomyelitis.

**Conclusions:**

The lack of scientific knowledge on hereditary sensory and autonomic neuropathy due to the rarity of the disease often results in a delay in diagnosis, which is of substantial importance for the prevention of many complications and symptoms. Interdisciplinary work of specialized medical and dental teams and development of a standardized treatment protocols are essential for the management of the disease. There are many knowledge gaps concerning the management of patients with hereditary sensory and autonomic neuropathy-VIII, therefore more research on an international basis is needed.

## Background

Hereditary sensory and autonomic neuropathy (HSAN) comprises a group of genetic disorders involving sensory and autonomic dysfunctions [1]. HSAN was classified into five main types [2]. Later, it was modified into subtypes [3–5] according to gene mutations, mode of inheritance, and clinical characteristics. HSAN types VI and VII were mentioned in the classification of Haga *et al*. [5]: Online Mendelian Inheritance in Man (OMIM) 614653 and 615548 respectively. HSAN type VIII (OMIM 616488) was recently characterized by Chen *et al*. [6] as a rare autosomal recessive disorder. Our patient’s family was included in their study. General characteristics, mode of inheritance, onset, and genes involved in each type of HSAN are presented in Table 1.

**Table 1:** Classification of recent types and subtypes of hereditary sensory (and autonomic) neuropathy

Types of HSAN	OMIM	Inheri.	Onset	Clinical characteristics	Somatosensory modalities	Sweating	Genes/ locus
HSAN-IA [2, 25, 28, 33, 34]	162400	AD	Mostly adolescence to adulthood	Hearing loss, loss of distal reflexes/distal muscle weakness, (no autonomic dysfunction)	Loss of pain and temperature sensation, lancinating pain	Normal	*SPTLC1/9q22.31*
HSAN-IB [2, 25, 28, 33–35]	608088	AD	Adulthood	Chronic cough, gastropharyngeal reflux, hearing loss, alacrima, impotence	Sensory loss, lancinating pain	Normal to mild distal hypohidrosis	*SPTLC1/3p24-p22*
HSAN-IC [2, 25, 28, 33, 34, 36]	613640	AD	Mostly adulthood	Ulcerative mutilations, variable distal motor involvement, distal muscle weakness, osteomyelitis	Loss of pain, lancinating pain, loss of temperature sensation in parts of the body, sensory loss in the upper and lower limbs	Normal	*SPTLC2/14q24.3*
HSN-ID [2, 25, 28, 33, 34, 37]	613708	AD	Adulthood	Ulcerative mutilations, trophic skin and nail changes, distal amyotrophy in the lower limbs	Distal sensory loss of the lower limbs	Normal	*ATL1/14q22.1*
HSN-IE [2, 25, 28, 33, 34, 38]	614116	AD	Adulthood	Ulcerative mutilations, hearing loss, dementia	Loss of all somatosensory modalities, lancinating pain	Normal	*DNMT1/19p13.2*
HSN-IF [2, 25, 28, 33, 34, 39]	615632	AD	Adulthood	No autonomic involvement, diminished tendon reflexes, painless ulceration of the feet	Distal sensory loss of the lower limbs	Normal	*ATL3/11q13.1*
HSAN-IIA [2, 25, 28, 33, 34, 40]	201300	AR	Childhood	Self-mutilation behavior resulting in extensive orofacial injuries, weakness, acropathy	Loss of pain, temperature and touch sensation, no autonomic dysfunction	Normal	*WNK1/12p13.33*
HSAN-IIB [2, 25, 28, 33, 34, 41]	613114	AR	Childhood	Ulcerative mutilation of hands, feet, and orofacial structures, osteomyelitis, urge incontinence	Impaired nociception	Hyperhidrosis	*FAM134B/5p15.1*
HSN-IIC [2, 25, 28, 33, 34, 42]	614213	AR	Childhood to adolescence	Ulcerative mutilation and orofacial injuries, absent deep tendon reflexes, minor distal weakness, distal numbness of the hands and feet	Impaired position vibration senses	N/A	*KIF1A/2937.3*
HSAN-IID [2, 4, 25, 28, 33, 34, 43]	243000	AR	Congenital or adolescence	Autonomic nervous dysfunction, hearing loss, hyposmia, bone dysplasia, orofacial self-mutilation injuries	Loss of pain and temperature sensation, hypogeusia	Hypohidrosis	*SCN9A/2q24.3*
HSAN-III [2, 26, 29, 33, 34, 44, 58 63–66]	223900	AR	Congenital	Profound autonomic dysfunction, vasomotor instability, absence of deep tendon reflexes, alacrima, impaired blood pressure regulation, failure to thrive, orofacial self-mutilation, absent fungiform papillae on the tongue, increased salivation, low caries index	Loss of pain and temperature sensation	Hyperhidrosis	*IKBKAP/9q31.3*
HSAN-IV [2, 8, 9, 11, 13, 14, 26, 28, 29, 34, 35, 44–51]	256800	AR	Congenital	Self-mutilation with orofacial injuries, deep tendon reflexes usually intact, recurrent fever, corneal lesions, mental retardation, recurrent infections, skin hyperkeratosis and fissuring, generalized tonic-clonic seizures	Loss of pain and temperature sensation	Hypohidrosis to anhidrosis	*NTRK1/1q23.1*
HSAN-V [2, 26, 29, 34, 35, 52]	608654	AR	Congenital	Similar to HSAN IV	Loss of pain and reduced thermal sensation, loss of deep pain perception	Normal to hypohidrosis	*NGFB/1p13.2*
HSAN-VI [33, 53]	614653	AR	Congenital	Lack of psychomotor development, autonomic abnormalities, absence of deep tendon reflexes, feeding and respiratory difficulties, neonatal hypotonia, alacrima, blotching	Loss of pain and temperature sensation	N/A	*DST/6p12.1*
HSAN-VII [33, 54, 55]	615548	AD	Congenital	Self-mutilation, painless fractures, delayed motor development, gastrointestinal dysfunction	Loss of pain sensation	Hyperhidrosis	*SCN11A/3p22.2*
HSAN-VIII [6, 30]	616488	AR	Onset in infancy	Self-mutilation behavior with orofacial injuries, painless fractures, skin and bone infections, corneal injuries, no mental retardation	Reduced pain and temperature sensation	Hypohidrosis	*PRDM12/9q34.12*
HSN with spastic paraplegia [33, 34]	256840	AR	Early childhood	Mutilation acropathy, septic paraplegia	Loss of all somatosensory modalities	Normal	*CCT5/5p15.2*

*AD* autosomal dominant, *AR* autosomal recessive, *ATL1* atlastin GTPase 1, *ATL3* atlastin GTPase 3, *CCT5* chaperonin TCP1 subunit 5, *DNMT1* DNA (cytosine-5-)-methyltransferase 1, *DST* dystonin, *FAM134B* family with sequence similarity 134 member B, *HSAN* hereditary sensory and autonomic neuropathy, *HSN* hereditary sensory neuropathy, *IKBKAP* inhibitor of kappa light polypeptide gene enhancer in B-cells kinase complex-associated protein, *Inheri.* mode of inheritance, *KIF1A* kinesin family member 1A, *N/A* not available, *NGFB* nerve growth factor (beta polypeptide), *NTRK1* neurotrophic tyrosine kinase-1 receptor, *OMIM* Online Mendelian Inheritance in Man, *PRDM12* PR domain-containing protein 12, *SCN9A* sodium channel, voltage gated type IX alpha subunit, *SCN11A* sodium channel, voltage gated, type XI alpha subunit, *SPTLC1*, serine palmitoyltransferase long chain base subunit 1, *SPTLC2*, serine palmitoyltransferase long chain base subunit 2, *WNK1* WNK lysine deficient protein kinase 1

HSAN-VIII is characterized by five main features: insensitivity to pain and thermal stimuli, self-mutilation behavior, altered sweat and tear formation, absence of corneal reflexes, and presence of repeated infections of the skin and bone [6]. The syndrome was confirmed in 21 patients [6] and 10 different homozygous mutations in the PR domain-containing protein 12 gene (*PRDM12*) were identified [6]. Mutations in the *PRDM12* gene in humans cause developmental defects in the sensory neurons, leading to loss of pain perception. Great loss of the small myelinated Aδ fibers occurred in patients with HSAN-VIII. Skin biopsies revealed that the peripheral terminals of unmyelinated C fibers were altered [6].

Patients carrying *PRDM12* mutations lack the sensation of acute pain and temperature. Thus, these patients have numerous injuries, which may lead to recurrent infections of skin and bones, and bone deformities later in life. In addition, they lack corneal reflexes, which leads to progressive corneal scarring. However, other senses like light touch, vibration, and proprioception are normal. The only autonomic dysfunction observed was the reduction in sweating and tears formation. Intellectual abilities in patients with HSAN-VIII are normal [6].

Insensitivity to pain leads to severe oral mutilations, such as tooth luxation, severe dental attrition, premature tooth loss, bite wounds, and ulcerations [7–9]. The tongue, followed by the lips, and the oral mucosa, are the most common sites of self-inflicted injuries [10, 11]. The diagnosis of HSAN is challenging due to its rarity, similarity in clinical presentation to other auto-aggression or self-mutilation diseases, and lack of simple diagnostic tests [12]. It is mainly confirmed by the clinical presentation, genetic analysis, pharmacological tests, and neuropathological examinations [13]. Management of patients affected by HSAN-VIII is complicated due to the patients’ lack of awareness and perception of pain.

We aimed to present the manifestations and dental management of a patient with HSAN-VIII harboring the homozygous mutation *c.516G>C* (*p. Glu172Asp*) in the *PRDM12* gene [6], who has been followed up in our clinic for 16 years. A review on PubMed library (January 1985 to December 2016) on patients with HSAN with oral manifestations was performed (Table 2).

**Table 2:** Literature review concerning oral manifestations associated with hereditary sensory (and autonomic) neuropathy

Year of pub.	Authors	Type	Gene	Country/ ethnic group	Ts	N	Age	G	General characteristics	Oral manifestations
2016	Eregowda *et al*. [56]	IV	*NTRK1*	India/ Indian	CR	1	11 y	F	Thermal insensitivity, anhidrosis, low intelligence, deformed interphalangeal joints of fingers, corneal scarring, skin infections, osteomyelitis	Oral self-mutilation, dental traumata
2015	Ravichandra *et al*. [13]	IV	*NTRK1*	India/ N/A	CR	1	7 y	F	Insensitivity to pain and temperature, anhidrosis, self-mutilation, preservation of other sensory modalities, recurrent fever	Orofacial self-mutilation, dental traumata
2015	Ashwin *et al*. [11]	IV	*NTRK1*	India/ N/A	CS	8	4–17 y	6 M 2 F	Insensitivity to pain, self-mutilation, recurrent fever, recurrent infection in the lower limbs	Oral self-mutilation
2015	Chen *et al*. [6]	VIII	*PRDM12*	Inter/ Inter	GA	21	3–40 y	13 M 8 F	Insensitivity to pain and temperature, hypohidrosis, self-mutilation behavior, skin and bone infections, painless fractures, corneal injuries, no mental retardation	Orofacial self-mutilation
2014	Özkaya *et al*. [57]	IV	*NTRK1*	Turkey/ N/A	CR	1	10 y	M	Recurrent fever, anhidrosis, ulcers on the skin, osteomyelitis, hyperkeratotic lesions on elbows and knees	Orofacial self-mutilation
2014	Guven *et al*. [44]	IV	*NTRK1*	Turkey/ Turkish descent	CS	2	1 y, 17 y	M	Insensitivity to pain and temperature, self-mutilation behavior, non-healing skin, ulcerations on the dorsum of the hands, anhidrosis, hypo- and hyper-pigmented skin	Orofacial self-mutilation
2013	Gao *et al*. [8]	IV	*NTRK1*	China/ N/A	CR	1	8 m	M	Recurrent fevers, anhidrosis, dry warm skin, congenital corneitis	Oral self-mutilation, dental caries, malocclusion, cleft palate
2013	Fruchtman *et al*. [58]	IV	N/A	Israel/ N/A	CS	30	1 m–15 y	16 M 14 F	Infections, fever, orthopedic lesions	Orofacial self-mutilation
2010	Hutton and McKaig [45]	V	N/A	UK/ N/A	CR	1	6 y	F	N/A	Orofacial self-mutilation
2010	Zilberman *et al*. [46]	III	N/A	Israel/ N/A	HA	17	N/A	N/A	N/A	Thicker enamel formation
2009	Neves *et al*. [9]	IV	*NTRK1*	Brazil/ N/A	CR	1	2 y	F	Unexplained fever episodes, anhidrosis, self-mutilation behavior, mental retardation	Oral self-mutilation
2009	Paduano *et al*. [59]	IV	N/A	Italy/ Italian descent	CR	1	8.11 y	M	Self-mutilation, recurrent fever, osteomyelitis	Oral ulcers, limited mouth opening
2008	Romero *et al*. [60]	IV	N/A	Spain/ N/A	CR	1	22 m	F	Self-mutilation, recurrent fever	Orofacial self-mutilation
2008	Singla *et al*. [47]	V	N/A	India/ Indian descent	CR	1	10 y	M	Insensitivity to pain, normal response to thermal stimuli, bilateral corneal opacities, hypoplasia of the nipples	Presence of severe maxillary ridge resorption, congenitally missing permanent teeth
2006	Butler *et al*. [25]	IV	*NTRK1*	Ireland/ N/A	CR	1	9 m	M	Self-mutilation injuries on wrist and feet, insensitivity to pain, normal reaction to thermal stimuli	Orofacial self-mutilation
2006	Schalka *et al*. [27]	IV	N/A	Brazil/ Caucasian	CR	1	16 m	F	Lack of painful stimuli, episodes of unexplained fever, hypohidrosis	Orofacial self-mutilation
2006	Siqueira *et al*. [48]	V	N/A	Brazil/ N/A	CS	2	22 y, 16 y	1 M 1 F	Insensitivity to pain, self-mutilation behavior	Orofacial self-mutilation
2003	Bonkowsky *et al*. [12]	IV	*NTRK1*	USA/ Northern European	CR	1	4 m	M	Hyperkeratosis on palms, skin fissuring	Orofacial self-mutilation
2002	Mass *et al*. [52]	III	N/A	Israel/ Ashkenazi-Jewish descent	CS	28	N/A	N/A	N/A	Low levels of mutans streptococci and lactobacilli in saliva, high salivary flow
2002	Wolf *et al*. [61]	III	N/A	Israel/ Ashkenazi-Jewish descent	CS	46	6–16 y	31 M 15 F	Impaired pain perception, skeletal deformities, small stature, failure to thrive, recurrent pneumonia, orthostatic hypotension	Progressive degeneration of tongue fungiform papillae and taste buds, impaired taste, excessive drooling, impaired swallowing
2000	Theodorou *et al*. [62]	IV	N/A	Greece/ N/A	CR	1	4 y	M	Insensitivity to pain, self-mutilation, bone fractures, anhidrosis, mental retardation	Orofacial self-mutilation
2000	Erdem *et al*. [49]	IV	N/A	Turkey/ N/A	CR	1	10 y	M	Acute tibia osteomyelitis, broken finger tips	Malformed oral configuration, orofacial self-mutilation
1999	Kim *et al*. [50]	IV	N/A	Korea/ N/A	CR	1	16 m	M	Self-mutilation, fever, anhidrosis, generalized tonic-clonic seizures	Orofacial self-mutilation
1998	Amano *et al*. [7]	IV	N/A	Japan/ Asian	CS	18	1–22 y	12 M 6 F	Self-mutilation behavior, insensitivity to pain, anhidrosis, infections, malnutrition	Orofacial mutilation, premature loss of teeth, intraoral scars and ulcers, severe bruxism, dental traumata, halitosis
1998	Rodd *et al*. [51]	II	N/A	UK/ Asian	CR	1	4 y	M	Sensory loss affecting all modalities of sensation predominantly involving the limbs, mutilation, anhidrosis, acropathy of finger tips and feet	Full-thickness loss of the tongue tip, tissue loss from the lower lip, loss of pain sensation
1998	Mass *et al*. [63]	III	N/A	Israel/ Ashkenazi-Jewish descent	CS	32	5.8–19.8 y	17 M 15 F	Decreased pain sensation, impaired temperature and blood pressure regulation, alacrima, absent tendon reflexes	Orofacial self-mutilation, dental traumata, low caries index, hypersalivation, absence of the fungiform papillae on the tongue
1996	Mass *et al*. [64]	III	N/A	Israel/ Ashkenazi-Jewish descent	CS	20	5–39 y	14 M 6 F	Decreased pain sensation, impaired temperature and blood pressure regulation, alacrima, absent tendon reflexes	Orofacial self-mutilation
1994	Mass and Gadoth [65]	III	N/A	Israel/ Ashkenazi-Jewish descent	CS	38	N/A	23 M 15 F	Decreased pain sensation, impaired temperature and blood pressure regulation, alacrima, absent tendon reflexes	Dental traumata
1992	Mass *et al.* [66]	III	N/A	Israel/ Ashkenazi-Jewish descent	CS	66	N/A	N/A	Decreased pain sensation, impaired temperature, impaired blood pressure regulation, absent tendon reflexes, alacrima	Orofacial self-mutilation, dental traumata, low caries index, hypersalivation, absence of the fungiform papillae on the tongue
1989	Kouvelas and Terzoglou [28]	IV	N/A	Greece/ N/A	CR	1	5.5 y	M	Insensitivity to pain, self-mutilation, fever, anhidrosis	Orofacial mutilation
1987	Brahim *et al*. [67]	IV	N/A	USA/ N/A	CR	2	11 y, 7 y	M	Self-mutilation, fever, anhidrosis, osteomyelitis	Orofacial mutilation
1986	Thompson *et al*. [68]	III	N/A	USA/ Caucasian	CR	1	31 y	M	Insensitivity to pain, blotching of skin, diminished lacrimation	Orofacial mutilation (including auto-extraction of teeth), diminished taste sensation
2016	Zhang *et al*. [30]	VIII	*PRDM12*	N/A	CS	5	23–57 y	4 M 1 F	Insensitivity to pain, normal neurological examinations and intellect, corneal abrasions, lack of tear production, recurrent infections, unexplained self-mutilation	Unexplained orofacial mutilation
Review articles										
2015	Haga *et al*. [5]	IV, V	*NTRK1, NGFB*	Japan/ N/A	RA	N/A	N/A	N/A	Repeated fractures, joint dislocations, arthritis, osteomyelitis, avascular necrosis, Charcot arthropathy	Oral self-mutilation (including auto-extraction of teeth)
2014	Kumar *et al*. [69]	IV	*NTRK1*	India/ N/A	RA	N/A	N/A	N/A	N/A	Orofacial self-mutilation, premature loss of teeth, osteomyelitis, fractures of the jaws
2013	Limeres *et al*. [26]	IV	N/A	Spain/ N/A	RA	N/A	N/A	N/A	N/A	Oral self-mutilation (including auto-extraction of teeth)
2012	Mass [70]	III	*IKBKAP*	N/A / N/A	RA	N/A	N/A	N/A	Insensitivity to pain and temperature, vasomotor instability, respiratory distress, orthostatic hypotension, insensitivity to hypoxia, decreased deep tendon reflexes, alacrima	Absence of fungiform papilla, dental traumata, orofacial self-mutilation, proportionally small jaws, crowding of teeth, low caries rate, hypersalivation, impaired taste sensation
2003	Nagasako *et al*. [71]	HSAN IV	N/A	USA/ N/A	RA	N/A	N/A	N/A	Insensitivity to pain, self-mutilation, painless fractures, fever, hypohidrosis	Orofacial self-mutilation

*CR* case report, *CS* case series, *F* female, *G* gender, *GA* genetics article, *HA* histological article, *IKBKAP* inhibitor of kappa light polypeptide gene enhancer in B-cells kinase complex-associated protein, *Inter* international, *m* month(s), *M* male, *N* number of patients, *N/A* not available, *NGFB* nerve growth factor (beta polypeptide), *NTRK1* neurotrophic tyrosine kinase-1 receptor, *PRDM12* PR domain-containing protein 12, *Pub*. publication, *RA* review article, *Ts* type of study, *y* year(s), UK United Kingdom, USA United States of America

## Case presentation

**Fig. 1:**
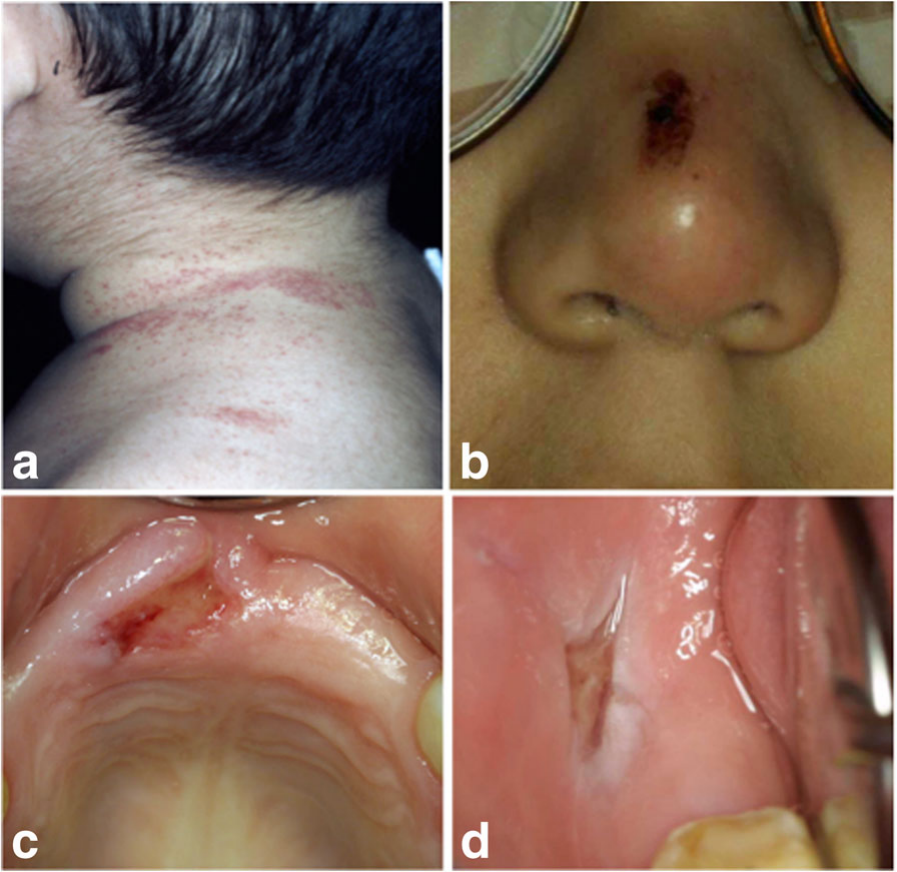
Representation of self-mutilation. **a**+**b** Extraoral self-mutilation. **c**+**d** Intraoral self-mutilation

**Fig. 2:**
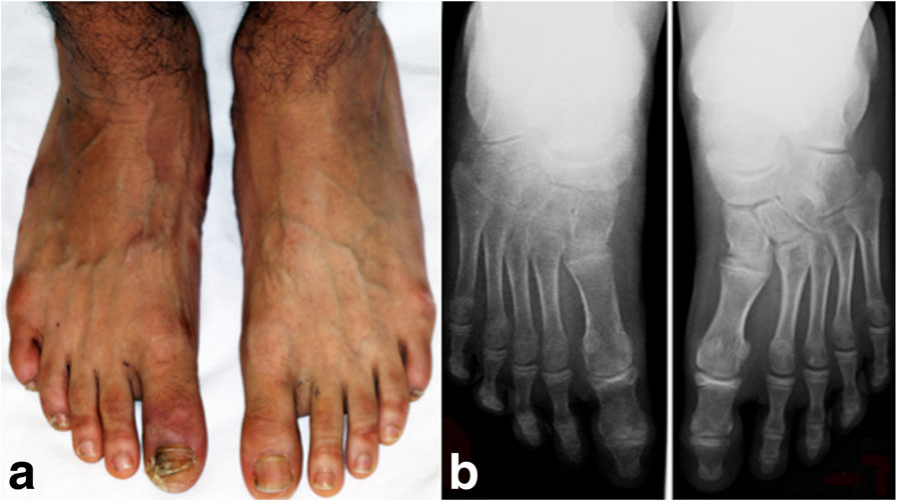
Bilateral bone deformity of the feet. **a** Clinical presentation of the deformed feet. **b** Radiographic presentation of the deformed feet

**Fig. 3:**
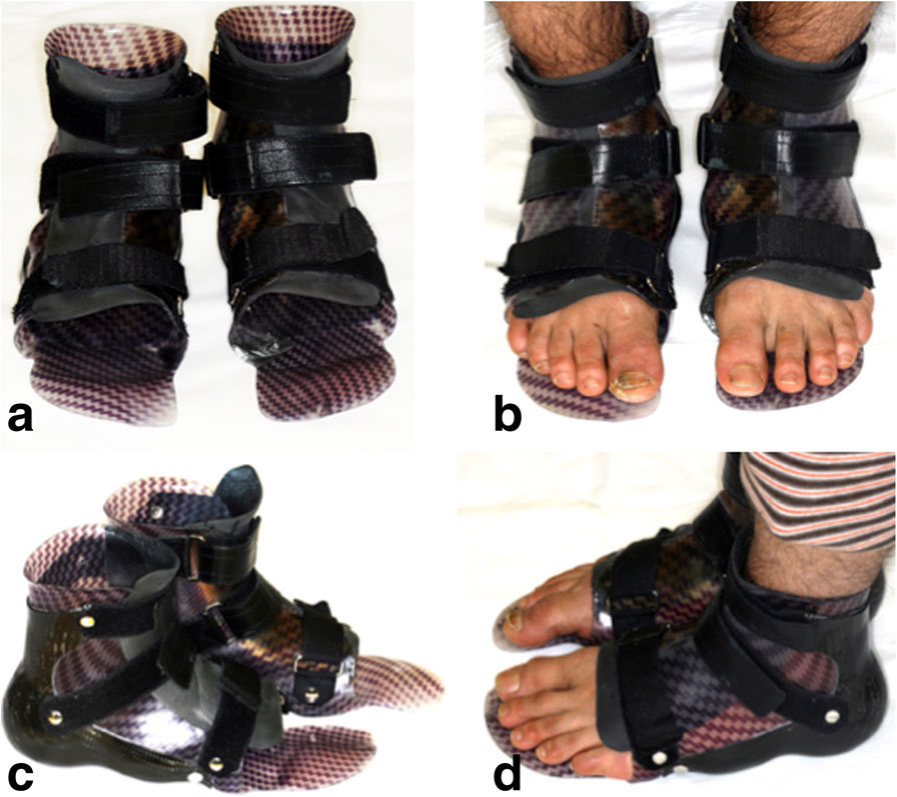
Orthopedic appliance used to support the patient’s feet during walking. **a**+**c** Orthopedic appliance. **b**+**d** Patient wearing his orthodontic appliance

An 8-month-old white boy of Turkish origin initially presented to the Department of Pedodontics, at Charité – Universitätsmedizin Berlin Hospital, due to an unexplained early loss of his primary lower central incisors. He was the first child of healthy consanguineous parents (second-degree relatives); their younger daughter was healthy. Our patient had multiple injuries on his face and body and in his oral cavity due to self-mutilation (Fig. 1). Further medical history revealed that he was born with a bilateral foot deformity (Fig. 2), which resulted in the mandatory use of an orthopedic appliance, enabling him to walk normally (Fig. 3). At the age of 6 years and 2 months, he had a fracture in the metatarsal bone, leading to bone necrosis. This resulted in the placement of bone plates and the use of a wheelchair for long walking distances. He had several accidents, such as severe burns from boiling water without feeling any pain.

**Fig. 4:**
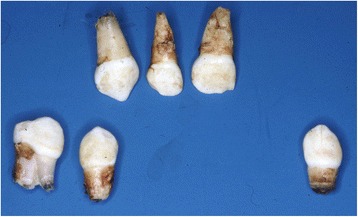
Auto-extracted teeth of the patient

Prior to the first visit to the Department of Pedodontics at the age of 8 months, he had lost both mandibular primary central incisors for unknown reasons only 3 months after they erupted. His mandibular left lateral incisor was loose (mobility, grade 2). In addition, his mandibular left primary second molar (75) showed signs of enamel hypoplasia. He experienced no pain or discomfort during the dental procedures. A year later, he presented at our department due to the further loss of ten of his primary teeth (Fig. 4). The early loss of so many teeth raised suspicion of a systemic disorder. He was referred to the Department of Human Genetics at Charité – Universitätsmedizin Berlin. The following differential diagnoses of auto-aggression syndromes were suspected: congenital insensitivity to pain and anhidrosis (CIPA), Smith–Magenis syndrome, Lesch–Nyhan syndrome, or pantothenate kinase-associated neurodegeneration (PKAN). In addition, the following systemic diseases, which might cause premature loss of teeth, were suspected: Langerhans cell histiocytosis, hypophosphatasia, and Papillon–Lefèvre syndrome. The diagnosis of CIPA syndrome was thought to be closest to his condition. All other suspected auto-aggression syndromes and systemic diseases were excluded based on blood tests, genetic diagnosis, and further clinical examination. However, after deeper investigations, the diagnosis HSAN-VIII was considered the definitive diagnosis of our patient.

**Fig. 5:**
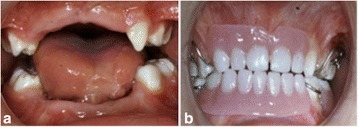
Prosthodontic treatment of the patient. (**a**+**b**) Intraoral pictures of patient without and with prostheses, respectively

Partial dentures for maxilla and mandible were constructed to prevent speech impairment and to enhance his lower facial height (Fig. 5). Due to his high caries activity, an intensive prophylaxis program with continuous follow-up was implemented to avoid further dental deterioration and improve his oral health status. Over the years, with the help of an interdisciplinary medical team and his parents, he has shown great cooperation and completely ceased any sort of self-mutilation behavior.

## Discussion

The pediatric dentist was the first to refer our patient to the human genetics department with the suspicion of HSAN syndrome, based on the premature loss of primary teeth and self-mutilation behavior. The initial diagnosis of our patient of CIPA or HSAN-IV was not confirmed by molecular analysis, since it did not detect a mutation in the neurotrophic tyrosine kinase-1 receptor gene (*NTRK1*), which is the receptor for nerve growth factor (NGF) related to CIPA syndrome [12]. Our patient harbored a homozygous mutation in the recently discovered gene *PRDM12* [6]. Therefore, HSAN-VIII was his final diagnosis. Deoxyribonucleic acid (DNA) sequencing of the parents confirmed the segregation of the mutation in the family. The mode of inheritance was autosomal recessive [6]. Self-mutilation behavior is one of the most outstanding characteristics of HSAN syndrome. However, it is also common in other auto-aggression diseases, which makes the diagnosis challenging. Smith–Magenis syndrome was a differential diagnosis concerning the self-inflicted injuries [14], but a causative *17p.11.2* microdeletion was excluded by fluorescence *in situ* hybridization. As for Lesch–Nyhan syndrome, patients have dystonia and ballism [15], which were not symptoms of our patient. Further analysis did not reveal defects in the hypoxanthine-guanine phosphoribosyltransferase (HPRT) enzyme, confirming the false diagnosis. PKAN is also characterized by dystonia and therefore was ruled out [16–19]. Blood tests excluded the systemic diseases of Langerhans cell histiocytosis and hypophosphatasia [20]. Hypophosphatasia was also excluded because of our patient’s normal total serum alkaline phosphatase activity [21–23]. Papillon–Lefèvre syndrome was not confirmed due to the absence of the diffuse palmoplantar hyperkeratosis and the progressive periodontitis [24]. Oral manifestations of HSAN are important, since they are one of the first complaints presented by affected patients. They can be detected early in life, starting with the eruption of the primary dentition [25]. Because of the variability and rarity of the clinical presentation of HSAN, no standard dental management protocols have been established. Patients with HSAN should be treated individually [26]. The dental treatment planning can be affected by several factors, such as age, mental development, and patient’s and parents’ compliance [27]. In the 1960s, the treatment approach for patients with HSAN was extraction of all primary teeth and construction of dentures in order to prevent self-mutilation. Nowadays, there are many dental treatment options for the prevention of self-mutilating behavior, varying from the elimination of sharp tooth surfaces by grinding or restoring them with resin composite, to the use of intraoral appliances such as mouthguards. Since the self-mutilation behavior of patients with HSAN-VIII starts in infancy, it may prove difficult to use intraoral appliances at that point. However, tooth extractions should be considered the last line of treatment. Early loss of teeth is one of the most frequent dental complications of HSAN. It is important to be able to deal with its consequences, such as speech impairment and increased incidence of malocclusions [25, 26, 28]. Professional dental cleaning, behavioral management, and routine check-up appointments were the cornerstones of our treatment plan. Prevention of dental disease is required in patients with HSAN, since caries progression and pulpal involvement can occur without causing any pain or discomfort. The parents of patients with HSAN play a crucial role in the management of the condition, since their psychological support is necessary to help the child understand his or her condition and prevent further injuries [27, 28]. The most critical phase of managing patients with HSAN would be building an understanding of the emotional experience of pain. A psychological approach should be introduced as early as possible [27]. Cognitive behavioral models for self-management and distress regulation have been proposed [29].

The literature search revealed that HSAN-IV (CIPA) is the most discussed form of HSAN in dentistry. Self-mutilation and auto-aggression are the first and most common clinical characteristics in all mentioned HSAN types (Table 2). The literature review results mainly consisted of case reports and case series, which is understandable due to the rarity of the syndrome. In contrast to our case report, a long follow-up period was not reported in the majority of publications. Our case report is, to the best of our knowledge, the first to discuss the oral manifestations and management of HSAN-VIII. Zhang *et al*. [30] also reported on the clinical characteristics of five patients with HSAN-VIII and was in line with Chen *et al*. [6]. The clinical characteristics described by Zhang *et al*. [30] that were found in all patients were: insensitivity to pain, normal neurological examinations and intellect, corneal abrasions, lack of tear production, recurrent infections, and unexplained oral self-mutilation (especially tongue injuries). There is a need for further dental and medical management solutions for these patients, as well as for well-educated practitioners [29]. There are many obstacles that have to be overcome since often there is a lack of resources for research and international collaboration and for accessible database and diagnostic and treatment tools. By expanding our knowledge on genetic and epigenetic factors that are critical for pain sensation, new fields are opened for therapeutic intervention in chronic and neuropathic pain conditions [6, 31, 32].

## Conclusions

HSAN-VIII is a rare, complex, recently identified condition mainly characterized by insensitivity to pain and thermal stimuli. The affected persons are vulnerable to various complications and in severe cases, self-mutilation can lead to death. Early identification of the disease is important to prevent all these consequences. The literature contains mainly case reports and case series of patients with HSAN, therefore, there are many knowledge gaps concerning preventive and therapeutic approaches. Treatment efficacy depends on educating the family and supporting the child psychologically. Moreover, an interdisciplinary treatment approach, in which there is medical and dental interdisciplinary cooperation, is required for such patients. A homozygous mutation of the *PRDM12* gene, which is responsible for the HSAN-VIII condition, was identified in our patient. Mutations in this gene cause developmental defects in sensory neurons before their transition to nociceptors.

## Abbreviations

CIPA: Congenital insensitivity to pain and anhidrosis
HSAN-VIII: Hereditary sensory and autonomic neuropathy type VIII
NGF: Nerve growth factor
OMIM: Online Mendelian Inheritance in Man
*NTRK1*: Neurotrophic tyrosine kinase-1 receptor
PKAN: Pantothenate kinase-associated neurodegeneration
*PRDM12*: PR domain-containing protein 12 gene
